# NT-proBNP, troponin I and troponin T are elevated in ARDS patients without structural heart disease: a single initial reading of cardiac markers is not different from serial daily readings

**DOI:** 10.1186/cc9551

**Published:** 2011-03-11

**Authors:** Y Nassar, D Monsef, S Abdelshafy, G Hamed

**Affiliations:** 1 Cairo University, Cairo, Egypt

## Introduction

Myocardial injury and cardiac marker elevation may occur in ARDS patients without a structural heart disease, which might affect cardiac markers [1,2].

## Methods

The study was conducted in Cairo University Hospital between 1 June 2008 and 1 April 2009. The inclusion criterion was any adult patient diagnosed to have ARDS according to the criteria of the American-European Consensus Conference of 1994. Exclusion criteria were any pre-existing structural heart disease, pulmonary embolism, atrial fibrillation, renal insufficiency, age <18. Plasma levels of cardiac markers NT-proBNP, troponin I and troponin T were measured on day 0 and on day 2 and day 7 of ARDS diagnosis. All patients benefited from mechanical ventilation with a lung-protective ventilation strategy according to the NHBLI ARDS Network Treatment Protocol.

## Results

The study comprised a total of 20 patients with mean age of 58.9 ± 20.69 years, 11 men versus nine women (*P *> 0.05). The ARDS aetiology was five (25%) patients due to sepsis, four (20%) due to pneumonia, three (15%) aspiration, three (15%) lung contusions due to road traffic accidents (RTA), two (10%) drug overdose, one (5%) burns, one (5%) pancreatitis, one (5%) drowning. NT-proBNP mean values were 8,903.3 ± 12,852.8 versus 6,083.6 ± 8,467.9 versus 9,914.8 ± 12,574.1 on day 0, day 2 and day 7, respectively (*P *> 0.05). Troponin I mean values were 3.0 ± 7.7 versus 2.2 ± 6.6 versus 1.5 ± 4.4 on day 0, day 2 and day 7, respectively (*P *> 0.05). Troponin T mean values were 0.3 ± 0.6 versus 0.6 ± 1.5 versus 0.5 ± 1.1 on day 0, day 2 and day 7, respectively (*P *> 0.05).

## Conclusions

ARDS patients with structurally normal hearts show persistent elevated levels of cardiac markers NT-proBNP, troponin I and troponin T over the first week with no significant change between levels of day 0, day 2 and day 7. A single reading of cardiac markers on any day of the first week of ARDS may not be different from serial daily readings.
